# Sexuelle und reproduktive Gesundheit: Datengrundlagen, Lebenslaufperspektiven und Anforderungen an Prävention und Versorgung

**DOI:** 10.1007/s00103-026-04215-9

**Published:** 2026-04-01

**Authors:** Sara Scharmanski, Ursula von Rüden

**Affiliations:** 1 Sexualaufklärung, Verhütung und Familienplanung, Bundesinstitut für Öffentliche Gesundheit (BIÖG), Maarweg 149–161, 50825 Köln, Deutschland; 2 Evaluation, Methoden und Forschungsdaten, Bundesinstitut für Öffentliche Gesundheit (BIÖG), Maarweg 149–161, 50825 Köln, Deutschland

Sexuelle Gesundheit ist ein zentrales Handlungsfeld der globalen Strategie der Weltgesundheitsorganisation (WHO) und wird wie folgt definiert: „Sexuelle Gesundheit ist ein Zustand des körperlichen, emotionalen, geistigen und sozialen Wohlbefindens in Bezug auf Sexualität und bedeutet mehr als nur das Fehlen von Krankheit, Funktionsstörungen oder Beeinträchtigungen. Sie erfordert eine positive und respektvolle Haltung gegenüber Sexualität und sexuellen Beziehungen sowie die Möglichkeit für lustvolle und sichere sexuelle Erfahrungen, frei von Unterdrückung, Diskriminierung und Gewalt. Damit sexuelle Gesundheit erreicht und erhalten werden kann, müssen die sexuellen Rechte aller Menschen anerkannt, geschützt und respektiert werden“ [1].

Die Fragen zur sexuellen Gesundheit sind entsprechend weitreichend: Sie betreffen Sexualität und Beziehungen, sexuelle Selbstbestimmung und Rechte, den Schutz vor sexualisierter Gewalt und sexuell übertragbaren Infektionen (STI). Im Sinne des menschenrechtsbasierten Ansatzes der WHO trägt ein positiver und respektvoller Umgang mit Sexualität, der frei von Zwang, Diskriminierung und Gewalt ist, zum allgemeinen Wohlbefinden und damit zur Lebensqualität der Bevölkerung bei. Sexuelle Gesundheit umfasst neben Selbstbestimmung, Bildung und Zufriedenheit auch die Entwicklung und das Ausleben sexueller Identität. Eng verbunden damit ist die reproduktive Gesundheit, die das Wohlbefinden in Bezug auf das Fortpflanzungssystem und dessen Funktionen sowie das Recht umfasst, frei und informiert über Fortpflanzung zu entscheiden.

Sexuelle und reproduktive Gesundheit sind zwar auf konzeptioneller Ebene häufig gemeinsam gerahmt („sexual and reproductive health“), aber nicht deckungsgleich: Sexuelle Aktivität dient meistens nicht der Reproduktion, während Reproduktion aufgrund des reproduktionsmedizinischen Fortschritts auch ohne Sexualität möglich ist.

Vor diesem Hintergrund bündelt das vorliegende Schwerpunktheft aktuelle sozialwissenschaftliche und interdisziplinäre Arbeiten zur sexuellen und reproduktiven Gesundheit in Deutschland. Ziel ist es, zentrale Datengrundlagen fortzuschreiben, neue Erkenntnisse zeitnah in die Fachöffentlichkeit zu transferieren und Impulse für Forschung, Prävention, Beratung, Versorgung sowie Sexualaufklärung und sexuelle Bildung zu geben.

Im ersten Teil stehen repräsentative Datengrundlagen und deren Fortschreibung im Mittelpunkt. *Halbach et al.* berichten Ergebnisse der „LIEBESLEBEN-Studie“ zur Bekanntheit der Impfung gegen humane Papillomviren (HPV) für Mädchen und Jungen in Deutschland. *Scharmanski und Dinger* stellen aktuelle Befunde zum Sexual- und Verhütungsverhalten Jugendlicher und junger Erwachsener aus der 10. Welle der repräsentativen Wiederholungsbefragung „Jugendsexualität“ vor. Beide Studien liefern aktuelle präventionsrelevante Erkenntnisse zur sexuellen und reproduktiven Gesundheit. Sie unterstreichen zugleich die Bedeutung zielgruppengerechter Gesundheitsinformation und -kommunikation und dienen der Weiterentwicklung von Prävention, Beratung und Sexualaufklärung.

Der zweite Teil widmet sich reproduktiver Gesundheit und Familienplanung. *Mannebach et al.* untersuchen in der Studienreihe „frauen leben 4“ die Entstehung kritischer Einstellungen zu hormonellen Kontrazeptiva und deren Auswirkungen auf die Verhütungspraxis. Der Beitrag macht deutlich, dass Verhütungsentscheidungen in einem Zusammenspiel aus Erfahrungen, Kommunikation, Beratung und gesellschaftlichen Narrativen entstehen und dass Entscheidungsunterstützung mehr umfasst als reine Wissensvermittlung. *Zitzmann* gibt einen Überblick über wissenschaftliche Erkenntnisse und Entwicklungen zur Kontrazeption für den Mann. Damit wird eine Perspektive gestärkt, die Fragen der geteilten Verhütungsverantwortung und der Erweiterung von Handlungsspielräumen in der Familienplanung berührt*. Böhm et al.* widmen sich den Rahmenbedingungen informierter Entscheidungen und interdisziplinärer Zusammenarbeit bei Schwangerschaftsabbrüchen. Der Beitrag zeichnet Strukturen und Prozesse nach, die Zugang, Qualität und Kooperationen in der Versorgung prägen. *Kavemann und Nagel* nehmen schließlich Elternschaft nach erlebter sexueller Gewalt in Kindheit und Jugend in den Blick und zeigen, wie Betroffene Schwangerschaft, Geburt und Elternsein erleben und welche Unterstützungsbedarfe daraus folgen.

Im dritten Teil stehen sexuelle Selbstbestimmung und der Schutz vor sexualisierter Gewalt im Mittelpunkt. *Dinger et al.* analysieren sexualisierte Gewalterfahrungen, Perspektiven von Bystandern (Dritten, die während der Gewalterfahrung anwesend sind oder davon erfahren) und Disclosure (Offenlegung sexualisierter Gewalterfahrungen) junger Menschen anhand aktueller Daten aus der „Jugendsexualitätsstudie“. Damit werden neben den Betroffenenerfahrungen auch soziale Kontexte sichtbar, in denen Offenbarungsprozesse und Unterstützung stattfinden*. Stehr und Wazlawik* untersuchen anschließend Unterstützungswege queerer junger Menschen bei sexuellen Grenzverletzungen und sexualisierter Gewalt. Der Beitrag nimmt Barrieren und Ressourcen in Hilfe- und Beratungsstrukturen sowie die Anforderungen an Sensibilisierung und Zielgruppenorientierung in den Fokus*. Pöge et al.* adressieren eine bislang selten quantitativ untersuchte Facette sexueller Gesundheit trans und nichtbinärer Menschen: sexuelle Ablehnungskompetenz als Voraussetzung dafür, unerwünschte sexuelle Handlungen ablehnen zu können.

Der vierte Teil führt sexuelle und reproduktive Gesundheit zusammen und zeichnet Implikationen für die Gesundheitskommunikation und sexuelle Bildung in analogen und digitalen Kontexten auf. *Watson et al.* ordnen internationale Erkenntnisse zur Sexualaufklärung 15 Jahre nach Veröffentlichung der „Standards for Sexuality Education in Europe“ (2010) ein. *Döring et al.* untersuchen mit einer Inhalts- und Qualitätsanalyse deutschsprachige Menstruationsvideos auf YouTube, Instagram und TikTok und zeigen auf, dass das Thema in sozialen Medien informations- und diskursorientiert verhandelt wird. Ebenfalls von *Döring et al.* stammt schließlich ein aktueller Beitrag zum Umgang Jugendlicher mit Online-Pornografie und den daraus resultierenden Bildungsanforderungen.

Über alle Beiträge hinweg wird deutlich: Sexuelle und reproduktive Gesundheit ist ein hoch relevantes, interdisziplinäres Public-Health-Feld. Auf Grundlage eines menschenrechtsbasierten Ansatzes und einer validen Datenbasis werden hier Versorgungs- und Beratungsstrukturen, Rechte- und Schutzperspektiven sowie Anforderungen an Bildung und Kommunikation zusammengeführt. Das vorliegende Schwerpunktheft bietet eine aktuelle, evidenzbasierte Grundlage. Es soll zu weiteren Forschungsvorhaben und Praxisentwicklungen anregen und dazu beitragen, Zugänge, Bedarfe und Wirksamkeit von Prävention, Beratung und Versorgung sowie Sexualaufklärung weiter zu stärken.
